# Endoscopic Surveillance in Inflammatory Bowel Diseases: Selecting a Suitable Technology

**DOI:** 10.3389/fmed.2022.855652

**Published:** 2022-03-30

**Authors:** Arianna Dal Buono, Roberto Gabbiadini, Federica Furfaro, Marjorie Argollo, Thaís Viana Tavares Trigo, Alessandro Repici, Giulia Roda

**Affiliations:** ^1^IBD Center, Department of Gastroenterology, Humanitas Research Hospital - IRCCS, Milan, Italy; ^2^IBD Center, Department of Gastroenterology, Universidade Federal de São Paulo, São Paulo, Brazil; ^3^Department of Biomedical Sciences, Humanitas University, Milan, Italy

**Keywords:** endoscopy, colorectal cancer, surveillance, chromoendoscopy, dysplasia, inflammatory bowel disease

## Abstract

In the treat-to-target era, endoscopy has become the backbone of the assessment of remission, defined as mucosal healing, in inflammatory bowel disease (IBD) patients. Current recommendations indicate that endoscopic procedures should be performed with high-definition white-light endoscopy (HD-WLE), as it guarantees the best possible visualization of the mucosa. With respect to endoscopic surveillance, the preventive strategy for dysplasia and colorectal cancer (CRC) in long-standing IBD, is the use of dye-chromoendoscopy (DCE), which enhances the mucosal pattern of the colonic walls. DCE has been established as the gold standard for dysplasia detection and is at present incorporated in all international guidelines. Over the past years, novel technologies, such as high-definition endoscopic imaging, and optical and digital enhancement tools have revolutionized the quality and level of fine details of vascular and mucosal patterns. These endoscopic images have the ambition to reflect histological changes for suspected neoplastic lesions and inflammation or healing and are emerging as potential alternatives to DCE. Indeed, the comparison of DCE with high-definition imaging is an open issue that deserves further investigation. We aimed to examine and summarize the technical aspects and the current evidence on endoscopic technologies with a specific focus on the surveillance in IBD patients.

## Introduction

Over the past years, the role of endoscopy in inflammatory bowel diseases (IBD) has been established in the diagnosis, monitoring, and surveillance (1, 2). The endoscopic evaluation allows to confirm the diagnosis of IBD, and the assessment of the extent and severity of the disease (1, 2). However, in the treat-to-target era, endoscopy is not a mere imaging technique any longer, but it has become the backbone of the assessment of remission. The endoscopic remission defined as mucosal healing (MH) has been proven to predict better long-term outcomes and has been incorporated as the principal target in the management of IBD (3, 4). Data on the prevention of bowel damage in the long-term, in patients achieving MH, are largely available both for Ulcerative Colitis (UC) and Crohn's Disease (CD) (3, 4), and have been endorsed by meta-analyses (5, 6).

As concerns technical aspects, current recommendations indicate that endoscopic procedures should be performed with high-definition white-light endoscopy (HD-WLE), as it guarantees the optimal visualization of the mucosa (1, 2). On the other hand, with respect to endoscopic surveillance as a preventive strategy for dysplasia and colorectal cancer (CRC), the use of dye-chromoendoscopy (DCE), which enhances the mucosal pattern through spraying topical dyes on the colonic walls, has been established as the gold standard for dysplasia detection (7–9).

Patients with long-standing (more than 8 years from the onset of symptoms) both extensive UC and colonic CD are at an increased risk of developing CRC compared with the general population, with a cumulative risk of 2% after 10 years, 8% at 20 years, and 18% at 30 years of disease (10). Among the established clinical factors that affect the cancer risk in IBD patients, the duration and extent of disease, chronic uncontrolled inflammatory activity, a concomitant diagnosis of primary sclerosing cholangitis (PSC), and a family history of CRC are included (11). On the other hand, due to improved therapies and increased integration of surveillance programs in the clinical practice, a decreasing risk of CRC in IBD patients has been observed in the recent years (12). Hence, an optimal surveillance colonoscopy is a crucial prevention, based on the detection of early colonic dysplastic lesions. Colonic precancerous lesions in patients with IBD are frequently flat and elusive, and during the colonoscopy, they can be easily missed especially when hidden behind folds, when there are regenerative patterns, scars, pseudopolyps, or actively inflamed mucosa.

As stated by the Surveillance for Colorectal Endoscopic Neoplasia Detection and Management in Inflammatory Bowel Disease Patients (SCENIC) consensus, HD-WLE is considered superior over standard definition WLE, and DCE should be preferred over WLE in the surveillance setting of IBD patients (8). According to SCENIC recommendations, whenever DCE cannot be performed, for instance due to disease activity or sub-optimal bowel preparation, random quadrant biopsies every 10 cm in the colon are advised (8). Recent novel technologies, such as optical and digital enhancement tools, have revolutionized the quality and level of fine details of vascular and mucosal patterns. These endoscopic images have the ambition to reflect histological changes in suspected neoplastic lesions and inflammation or healing, and are emerging as potential alternatives to DCE in the field of surveillance programs. The optimal endoscopic technique to detect dysplasia in IBD is still a matter of debate. In this review, we aim to examine and summarize the technical aspects and the current evidence on endoscopic technologies with a specific focus on the surveillance, in terms of dysplasia detection and characterization, in IBD patients.

## Methods

PubMed/MEDLINE databases were searched up to December 2021 to identify relevant studies investigating the accuracy of endoscopic techniques in dysplasia detection and characterization in IBD patients. The following text words and corresponding Medical Subject Heading/Entree terms were used: “surveillance,” “dye chromoendoscopy,” “virtual chromoendoscopy,” and “endocytoscopy,” individually and in combination with “dysplasia,” “inflammatory bowel disease(s),” “IBD,” “ulcerative colitis,” and “Crohn's disease.” No publication date restrictions were applied. Articles were included in this review based on their relevance, and additional publications were identified through their reference lists.

## Dye-Chromoendoscopy

**Figure 1:**
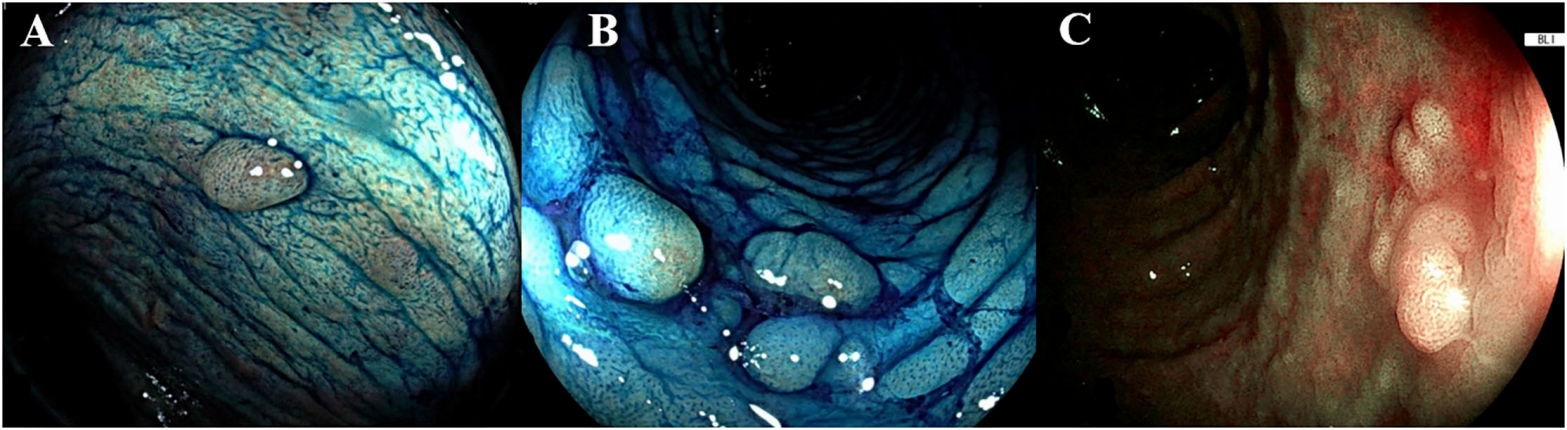
Endoscopic appearance of dye-chromoendoscopy and virtual chromoendoscopy. **(A,B)** The dye is segmentally applied to the colon every 20 to 30 cm with rotational movements of the endoscope, after spraying the dye, once the excess liquid has been suctioned, the mucosa is carefully examined. The colic mucosa presents a regular pattern, the presence of inflammatory pseudo-polyps is enhanced (pit pattern Kudo I-II). **(C)** A sporadic flat lesion with adenomatous appearance (pit pattern Kudo IIIS) in a UC patient, characterized through the blue laser image (BLI, Fujifilm, Japan). According to Kudo classification type I pits appear roundish; type II pits appear as stellar or papillary; type III-s pits are small roundish, tubular pits and type III-L are larger roundish and tubular; type IV pits appear as branch-like or gyrus-like while type V pits are non-structured. The normal mucosa, hyperplastic lesions and inflammatory polyps are comprehended in type I and II, whereas the pit pattern of the classes III-V is considered neoplastic.

The European Society of Gastrointestinal Endoscopy (ESGE) and the SCENIC consensus recommend the routine use of 0.1% methylene blue or 0.1–0.5% indigo carmine pancolonic chromoendoscopy with targeted biopsies for neoplasia surveillance in patients with long-standing IBD. Indigo carmine is a non-absorbed dye, while methylene blue is an absorbed dye, which can be applied using different concentrations depending on whether the purpose is detection (lower concentrations) or characterization of lesions (higher concentrations). The dye solution can be distributed with a catheter spray or a pump jet. All guidelines indicate that DCE with targeted biopsies should be favored over random biopsies (1, 2, 8, 9). Figure 1 shows the typical appearance of colonic mucosa after the application of methylene blue.

As demonstrated by a recent observational study, the most frequent causes of an unsuccessful DCE were a poor bowel preparation, active inflammation, and/or the presence of pseudopolyps (13) (Figure 1). Moreover, the non-adherence to a clear liquid diet the day before DCE compromised the successful completeness of DCE, compared with previous identified risk factors for inadequate bowel preparation (i.e., older age, history of diabetes mellitus, timing of split dose, narcotic use, and constipation), that were not associated with the ability of performing DCE (13).

DCE by appropriately trained operators, in all cases of quiescent disease and satisfactory bowel preparation, is preferred over the traditional non-targeted four-quadrant biopsies (9).

A properly trained operator is experienced in inspection, advanced imaging, and characterization of colonic lesions. Favorably, she/he is also trained in therapeutic endoscopic resection techniques such as endoscopic mucosal resection (EMR) and endoscopic submucosal dissections (ESD).

Few data are available on the characteristics of a complete training in DCE, and currently, there is no quality index to establish the expertise of endoscopists in performing DCE. However, according to the European Society of Gastrointestinal Endoscopy (ESGE), it is suggested that the endoscopist, in order to achieve the proper competence, should attend an onsite training of at least a week with an expert operator in optical diagnosis of IBD (14). The indicated number for adequate self-learning is at least 20 DCE surveillance procedures in IBD patients (14).

As concerns the performance of DCE, this technique has been shown to improve dysplasia detection by 4-fold, with less biopsies (15–18). In the first randomized controlled study by Kiesslich et al., as compared with WLE random biopsy sampling, DCE showed a significant 3.2-fold increase in the total detected neoplastic lesions in UC patients (16). Data from the prospective study by Picco et al. showed that DCE with indigo carmine in the surveillance of long-standing UC was superior in terms of dysplasia detection in comparison to WLE (21.3 vs. 9.3%, *p* = 0.007) (17). In a recent trial where IBD patients were randomized either to HD-DCE with indigo carmine or to HD-WLE (*n* = 152 and *n* = 153, respectively), dysplastic lesions were significantly more frequently detected in the HD-DCE arm (17 versus 7, respectively; *p* = 0.032) (18).

The SCENIC meta-analyses, which included eight studies, confirmed that DCE detected a significantly higher number of dysplastic lesions compared with standard-definition WLE (SD-WLE) [relative risk (RR) = 1.8, 95% confidence interval (CI): 1.2–2.6; and absolute risk increase = 6%, 95% CI: 3%−9%] (8).

Table 1 summarizes the available studies on DCE, WLE, and virtual chromoendoscopy (VCE) in the field of surveillance in IBD.

**Table 1:** Studies investigating dysplasia detection rate in IBD with different endoscopic technologies.

**Reference**	**Year**	**Study design**	***N***	**Type of endoscopy**	**Investigated outcome**
Kiesslich et al. (16)	2003	Randomized clinical trial	263	DCE vs. WLE	Dysplasia detection rates
Picco et al. (17)	2013	Prospective, non-randomized	75	DCE vs. WLE	Dysplasia detection rates and interobserver variability in the detection of dysplastic lesions
Alexandersson et al. (18)	2020	Randomized clinical trial	305	HD-DCE vs. HD-WLE	Dysplasia detection rates
Iacucci et al. (19)	2018	Randomized clinical trial	270	iSCAN vs. DCE vs. HD-WLE	Detection of colonic lesions
Bisschops et al. (20)	2018	Randomized clinical trial	131	NBI vs. DCE	Performance of DCE and VCE for the detection of neoplastic lesions
Efthymiou et al. (21)	2013	Prospective, non-randomized	44	NBI vs. DCE	Diagnostic yield of each modality for dysplastic lesions
Pellisé et al. (22)	2011	Prospective, randomized, crossover study	60	NBI vs. DCE	Detection of colonic lesions
Iannone et al. (23)	2017	Meta-analysis	1,500	DCE vs. SD-WLE/HD-WLE/NBI	Dysplasia detection rates
Kandiah et al. (24)	2021	Randomized clinical trial	188	iSCAN vs. HD-WLE	Dysplasia detection rates

*IBD, inflammatory bowel disease; DCE, dye-chromoendoscopy; WLE, wight light endoscopy; HD, high definition; NBI, narrow bad imaging*.

Necessarily, chromoendoscopy considerably increases the duration of colonoscopy (mean of 11 min, range 9–12 min) (8).

It appears clear that most of the available randomized clinical trials (RCTs) compared DCE with SD-WLE rather than with HD-WLE. Indeed, HD-WLE has been demonstrated to be not inferior to DCE for the detection of colonic dysplasia in a recent RCT, where HD-WLE alone was sufficient for detecting dysplasia, adenocarcinoma, or all neoplastic lesions (19). The comparison of DCE with high-definition imaging is an open issue that deserves further investigation.

## Virtual Chromoendoscopy

The currently available international guidelines instruct on the use of virtual chromoendoscopy (VCE) as a suitable alternative, due to insufficient evidence to recommend it as preferred method (9, 25). Initial studies for dysplasia detection in IBD have explored VCE. The technologies available in the market include the optical diagnosis narrow band imaging (NBI) (Olympus, Japan) that uses only wavelengths absorbed by hemoglobin for maximizing the contrast; the optical enhancement iSCAN (iSCAN, Pentax, Japan) that digitally adds blue color to relatively dark areas; the blue laser image (BLI) (Fujifilm, Japan) that can be used for evaluating both the microvessels and the mucosa; the linked color imaging (LCI) (Fujifilm, Japan) that expands the color range between red and white, enhancing slight mucosal differences in conditions of inflammation and cancer; and the flexible imaging color enhancement (FICE) (Fujinon) that selectively emphasizes certain ranges of wavelength. Through a digital post-processing of the endoscopic images, the VCE systems enhance the details of tissue surface and yearn to reflect histology with increased accuracy.

Among the available studies, a multicenter prospective study that randomized 66 patients in the DCE arm and 65 patients in the NBI arm, revealed no significant difference in neoplasia detection between the two techniques (20). In details, the mean number of neoplastic lesions per colonoscopy were 0.47 and 0.32, respectively (20). The dysplasia detection rate did not significantly differ between the two groups (21.2% with DCE, 21.5% with NBI; *p* = 0·964). However, in the DCE group, an average of additional 7 min to procedure time was reported (20). Two previous studies examining HD-NBI have reported the non-inferiority of HD-VCE over HD-DCE (21, 22).

Iacucci et al. compared HD-WLE with HD-iSCAN and HD-DCE, in a randomized non-inferiority trial (*n* = 270), and found no significant difference in the detection rates of neoplastic lesions between the techniques (19). Table 1 elucidates the characteristics of the above-mentioned studies.

A recent meta-analysis including 10 RCTs confirmed that DCE was able to identify more dysplastic lesions as compared with SD-WLE (RR = 2.12; 95% CI: 1.15–3.91). However, no statistically significant difference was observed between DCE and HD-WLE (RR = 1.42; 95% CI: 0.80–2.52) or NBI (RR = 1.05; 95% CI: 0.64–1.71) (23).

Data from the newly published VIRTUOSO trial, enrolling 188 IBD patients, which were randomized either to VCE (iSCAN, *n* = 94) or HD-WLE (*n* = 94), reported no significant difference between the two techniques for neoplasia detection (24). The dysplasia detection rates were assessed as 24.2 and 14.9% for HD-WLE and VCE, respectively, with no statistical significance (*p* = 0.14) (24). The authors observed similar withdrawal times in both arms of the study (median of 24 vs. 25.5 min for HD-WLE and VCE, respectively) (24).

Finally, no published data about either BLI or LCI to detect colonic lesion in patients with IBD are available yet.

## Characterization of Colonic Lesions

Since the SCENIC consensus, the term “dysplasia-associated lesion or mass” has been abandoned, with the recommendation to distinguish dysplasia as invisible or visible. In accordance with the knowledge development in endoscopy, the modified Paris classification was introduced to describe the morphology of the lesion and the border as regular or irregular, with or without ulceration (26). According to the Paris classification, colonic lesions are divided into polypoid and non-polypoid (if it protrudes <2.5 mm into the lumen). The polypoid lesions are then classified as pedunculated (with a stalk) or sessile (with a large base). Within the non-polypoid lesions, a distinction can be made between superficial elevated, flat, and depressed. Once morphology has been described, the operator inspects the surface pit pattern of the lesion, which can be described through Kudo's pit pattern classification. This classification comprises six categories (I, II, IIIS, IIIL, IV, and V) (27). The neoplastic Kudo pit pattern IIIS-IIIL-IV-V was found to be a significant predictor of dysplasia in patients with IBD (19). Further studies reported the same association, thereby making the neoplastic Kudo pit pattern (i.e., IIIS-IIIL-IV-V) one of the four main predictors of colonic dysplasia (28). In Figure 1, an example of the characterization of colonic lesions according to Kudo's pit pattern is shown.

It is known that the Kudo's pit pattern classification without magnification might have some limitations, especially in those IBD patients with a regenerative hyperplastic or villous mucosal appearance (29). Indeed, it has been demonstrated that in UC patients, on adding magnification, the pit pattern classification well-correlated with the histopathological diagnosis of low-grade and high-grade dysplasia and *in situ* carcinoma (30). Moreover, magnifying endoscopy allows to distinguish the margins of lesions from the surrounding mucosa and the differentiation between deep and superficial submucosal invasion in very suspicious lesions (31).

Lately, a new classification for IBD lesions has been introduced, which is the Frankfurt Advanced Chromoendoscopic IBD LEsions (FACILE) classification (32). This was developed by international experts and fully validated. The FACILE classification completely abandons the Kudo's pit pattern (32). Four characteristics, predicting neoplastic histology are incorporated in the FACILE classification: the morphology (i.e., nonpolypoid or polypoid), the irregular surface and vascular, and any sign of inflammation within the studied lesion (32). Furthermore, the authors demonstrated that trainees without endoscopic expertise could significantly ameliorate their ability in lesions' characterization with FACILE, with a sensitivity and an accuracy of 80% and 77%, respectively, after training (*p* < 0.001) (32).

Endocytoscopy, based on the principle of contact light microscopy providing real-time ultra-magnified images, has been more extensively investigated in the assessment of inflammation, but appears as a promising technology also in the field of colorectal lesions, especially in their characterization (33, 34). Endocytoscopy has been described in the setting of IBD-associated dysplasia in several case reports, thereby suggesting its future applicability (33, 34). It appears reasonable to expect that the use of endocytoscopy in IBD might potentially close the gap between endoscopic pit pattern diagnosis and histologic assessment.

## Conclusions and Future Perspectives

This review elucidates the current evidence on the performance and accuracy of the available endoscopic techniques and technologies in the field of CRC surveillance in IBD. It is known that DCE in comparison to SD-WLE improves the dysplasia detection by 4-fold, without the need of the four-quadrant random biopsies per each colonic tract (15–18).

As mentioned above, the published RCTs compared DCE with SD-WLE rather than with HD-WLE. The evaluation of the accuracy of DCE compared with high-definition endoscopy will be certainly addressed by dedicated studies in the immediate future. Importantly, so far, the non-inferiority of HD-WLE to DCE has been already reported, and based on these data, HD-WLE alone can be considered appropriate and satisfactory as concerns the detection of dysplasia (19).

An important matter of debate is the registered difficulty of adopting DCE into routine clinical practice. This might be due to the absence of properly trained and expert endoscopists and the low confidence of the operators in interpreting the DCE images. However, the mainly addressed cause is undoubtedly the additional time needed to perform a good quality DCE procedure. When comparing the usual colonoscopy with multiple non-targeted biopsies, chromoendoscopy considerably increases the duration of colonoscopy, with a mean of 11 min longer duration (range 9–12 min) (8).

This key issue represents the rational of the latter studies investigating VCE as an alternative method for CRC surveillance in IBD patients. If further multicenter studies and RCTs confirm the non-inferiority of HD-VCE over HD-DCE, and eventually its superiority in terms of time consumption and patients' tolerance, it might turn into an alternative procedure used by experienced IBD endoscopists in the coming years.

Moreover, surveillance with random biopsies results in extremely low dysplasia detection rates (24, 35). Indeed, the dysplasia detection of random four-quadrant biopsies ranges from 0/924 to 1/6,751 (24, 35).

Nevertheless, we agree with the scientific position that random biopsies should still have a role, always in association with DCE, in all IBD patients with a personal history of neoplasia/dysplasia, concomitant PSC, and/or a tubular appearance of the colon during colonoscopy (36, 37). Still, more data are needed on the true utility of random biopsies in these categories of IBD patients.

Lastly, artificial intelligence (AI)-based detection systems and computer-assisted diagnosis (CAD) systems are under increasing employment and development in endoscopy including IBD endoscopy (38). In this respect, the very first cases of AI used for the detection of dysplasia in patients with IBD have been recently reported (39). The application of AI in IBD endoscopy represents an appealing field for the future research.

Patients with IBD are at increased risk of developing colonic dysplasia and CRC (10). Endoscopic surveillance is demonstrated to significantly reduce CRC development and CRC-associated death, and increases the detection of early-stage CRC in IBD patients (*p* < 0.01) (40). As far as we are concerned, the best strategy to reduce this risk involves both the best quality surveillance colonoscopy method and an optimal medical control of disease activity through targeted therapies.

## Author Contributions

GR is the guarantor of the article, conceived the subject of the article, contributed to the critical interpretation, and supervised the project. AD and RG performed the research. AD wrote the manuscript. MA, TT, FF, and AR critically reviewed the content of the article. All authors approved the final version of the manuscript.

## Conflict of Interest

MA, Speaker, Congress Assistance and Advisory Board for Janssen, Takeda, Abbvie, Pfizer, and Sandoz. The remaining authors declare that the research was conducted in the absence of any commercial or financial relationships that could be construed as a potential conflict of interest.

## Publisher's Note

All claims expressed in this article are solely those of the authors and do not necessarily represent those of their affiliated organizations, or those of the publisher, the editors and the reviewers. Any product that may be evaluated in this article, or claim that may be made by its manufacturer, is not guaranteed or endorsed by the publisher.
